# Angiotensin receptor blockers are associated with lower mortality than ACE inhibitors in predialytic stage 5 chronic kidney disease: A nationwide study of therapy with renin-angiotensin system blockade

**DOI:** 10.1371/journal.pone.0189126

**Published:** 2017-12-07

**Authors:** Chih-Ching Lin, Yu-Te Wu, Wu-Chang Yang, Min-Juei Tsai, Jia-Sin Liu, Chi-Yu Yang, Szu-Yuan Li, Shuo-Ming Ou, Der-Cherng Tarng, Chih-Cheng Hsu

**Affiliations:** 1 Division of Nephrology, Department of Medicine, Taipei Veterans General Hospital, Taipei, Taiwan; 2 School of Medicine, National Yang-Ming University, Taipei, Taiwan; 3 Sungho Clinic, Ho-Ho-Ho Health Manage System, Taoyuan, Taiwan; 4 Division of Nephrology, Department of Medicine, Landseed Hospital, Taoyuan, Taiwan; 5 Department of Medicine, Chang-Hua Hospital, Ministry of Health and Welfare, Changhua, Taiwan; 6 Institute of Population Health Sciences, National Health Research Institutes, Zhunan, Taiwan; 7 Department of Health Services Administration, China Medical University, Taichung, Taiwan; 8 Department of Family Medicine, Min-Sheng General Hospital, Taoyuan, Taiwan; The University of Tokyo, JAPAN

## Abstract

Dual renin angiotensin system (RAS) blockade using angiotensin-receptor blockers (ARBs) in combination with angiotensin converting enzyme inhibitors (ACEIs) is reported to improve proteinuria in both diabetic and non-diabetic patients. However, its renoprotective effect and safety remain uncertain in patients with advanced chronic kidney disease (CKD). From January 1, 2000 through June 30, 2009, we enrolled 14,117 pre-dialytic stage 5 CKD patients with serum creatinine >6mg/dL and hematocrit <28% under the treatment with erythropoiesis stimulating agents and RAS blockade. We used Cox proportional hazards regression models to estimate the hazard ratios (HRs) against the commencement of long-term dialysis and all-cause mortality for ACEI/ARB users. Over a median follow-up of 7 months, 9,867 patients (69.9%) required long-term dialysis and 2,805 (19.9%) died before progression to end-stage renal disease requiring dialysis. In comparison with the ARB-only users, dual blockade with ACEIs and ARBs was associated with a significantly higher risk of (1) death in all CKD patients (HR = 1.49, [95%CI, 1.30–1.71]; P = 0.02) and in diabetic subgroup (HR = 1.58, [95%CI, 1.34–1.86]; P = 0.02); (2) composite endpoint of long-term dialysis or death in diabetic subgroup (HR = 1.10, [95%CI, 1.01–1.20]; P = 0.04); (3) hyperkalemia-associated hospitalization in non-diabetic subgroup (HR, 2.74, [95%CI, 1.05–7.15]; P = 0.04). However, ACEIs users were associated with higher mortality than ARBs users in all CKD patients (HR = 1.17, [95%CI, 1.07–1.27]; P = 0.03) and in diabetic subgroup (HR = 1.32, [95%CI, 1.18–1.48]; P = 0.03). Monotherapy of RAS blockade, especially ARB, is more effective and safer than dual RAS blockade in pre-dialytic stage 5 CKD patients.

## Introduction

Angiotensin-converting enzyme inhibitor (ACEI) or angiotensin II receptor blocker (ARB) has been prescribed worldwide to improve proteinuria and delay the progression of chronic kidney disease (CKD) in both diabetic and non-diabetic patients. Several investigations have documented its benefit for renal protection to the patients with early CKD (serum creatinine level: 1.5–3.0 mg/dl)[1, 2] and non-diabetic stage 4 CKD (glomerular filtration rate:15–29 ml/min/1.73m^2^ or serum creatinine level: 3.0–5.0 mg/dl).[3] To explore whether ACEI/ARB therapy is the same effective to those patients with advanced CKD at the pre-dialytic stage, our task group developed a national-wide retrospective study by including all CKD patients diagnosed between January 1, 2000 and June 30, 2009 in Taiwan, who had serum creatinine level >6 mg/dl and hematocrit level <28%, and could receive erythropoiesis-stimulating agent (ESA). Among 28,497 advanced CKD patients, 14,117 ACEI/ARB users, as compared with non-users, showed to run a significantly lower risk of long-term dialysis (HR, 0.94 [95% CI, 0.91–0.97]) and the composite outcome of long-term dialysis or death (0.94[0.92–0.97]).[4] Thus, the survival benefit of ACEI or ARB therapy can persist throughout the whole CKD stage, even in pre-dialytic patients.

Previous investigations have disclosed that dual renin angiotensin system (RAS) blockade (combination therapy with an ACEI and an ARB) is more effective in proteinuria reduction, which may provide additional cardiovascular or renoprotective benefit, than either drug alone in renal disease.[5] However, in the Ongoing Telmisartan Alone and in Combination with Ramipril Global Endpoint Trial (ONTARGET), the authors found that combination therapy with an ACEI and an ARB, compared with monotherapy, did not provide more cardiovascular or renal benefits but increased risk of hyperkalemia and acute kidney injury in persons running an increased cardiovascular risk.[6] Another recent meta-analysis for patients with early CKD (stage 1–3) showed no significant difference, either, between dual ACEI plus ARB combination therapy and monotherapy in reducing mortality risk or delay ESRD development.[7] However, investigation focusing on the safety and effectiveness of dual RAS blockade in advanced CKD patients, especially at pre-dialytic stage, is lacking. To bridge the gap in the transition from CKD to ESRD, we assessed the association of the choice of treatment (dual RAS blockade *vs* monotherapy) with the risk of long-term dialysis and/or death in this nationwide, large cohort of patients with pre-dialytic stage-5 CKD who had hypertension and anemia, and were treated with ESAs.

## Materials and methods

### Data source

The present study used data from Taiwan’s National Health Insurance (NHI) Research Database which was launched in 1995, managed and released to the public by the National Health Research Institute of Taiwan, and up to the present covers more than 99%, approximating 23 million, of the residents in Taiwan. This mandatory universal program offers all their medical records, including date of birth, sex, diagnostic codes, medical procedure and prescription of drugs. Diseases are coded according to the 2001 International Classification of Diseases, ninth revision, Clinical Modification (ICD-9-CM). Any information that would expose the identities of individual patients is de-identified. Having been taken as the primary source for several published studies, NHIRD has also had the accuracy of diagnoses be repeatedly validated). This study was approved by the Institutional Review Board of Taipei Veterans General Hospital.

### Study design

This nationwide, retrospective cohort study was performed in Taiwan to determine the association between ACEI/ARB usage and the prognosis of advanced CKD. Patients with a primary diagnosis of CKD (ICD-9 codes 016.0, 042, 095.4, 189, 223, 236.9, 250.4, 271.4, 274.1, 403–404, 440.1, 442.1, 446.21, 447.3, 572.4, 580–589, 590–591, 593, 642.1, 646.2, 753, and 984) subjected to ESA treatment combined with RAS blockade agent (ACEI or ARB) from January 1, 2000 through June 30, 2009 were enrolled into this study. The National Health Insurance has set the level of serum creatinine >6 mg/dl and hematocrit <28% as the indication for the use of ESA. According to the national report of the Department of Health, 85% of the patients with stage 5 CKD were subjected to ESA treatment if they were not on the list requiring renal replacement therapy. Since the median hematocrit level reported at the initiation of dialysis was 24.4% in an interquartile range from 20.6% to 27.5% in Taiwan, the cohort of the subjects selected in the current study should therefore be the most representative of patients with pre-dialytic stage 5 CKD. The use of ACEI/ARB was defined as prescription within 90 days after the first ESA treatment. To prevent immortal date bias, the 91th day after the first ESA treatment was re-defined as the index date. Patients who did not receive ACEI/ARB, who were younger than 20 years or older than 100 years of age, or who had received renal replacement therapy (including dialysis and renal transplantation) before ESA treatment were excluded from this study.

Totally 24,765 patients were included in the current study. The subjects who had received ACEI only, ARB only, or the combined therapy with ACEI plus ARB within 90 days after the first ESA prescription were defined as ACEI group, ARB group, or ACEI +ARB group accordingly. The remaining patients who took both ACEI and ARB without combination during this period were defined as ACEI/ARB group. Analyses were all conducted on an intention-to-treat basis according to the patients’ initial assignment regardless any subsequent changes in their ACEI/ARB regimen.

### Baseline characteristics

Baseline demographic characteristics, including age, gender, comorbidity, geographic location, nephrologist visits and medications were extracted and recorded. Nephrologist visits and comorbidities, including diabetes mellitus, stroke sequela, coronary artery disease and cancer, were defined as record within 3 years before the index date. Charlson comorbidity index (CCI) scores were used to determine overall systemic health. Each increase in the CCI score was associated with a stepwise increase of cumulative mortality. Anti-hypertensive agents other than ACEI/ARB analyzed in this study included diuretics, α-blockers, β-blockers, and calcium channel blockers. Medications that may potentially influence potassium balance were also excluded, e.g. Calcium polystyrene sulfonate, Sodium polystyrene sulfonate and sodium bicarbonate.

### Renal outcome and mortality

The observation period started from the index date till the patients died or began long-term dialysis, or December 31, 2009, whichever occurred earlier. The renal outcome was defined as the status when the patients entered long-term dialysis. The composite outcome of long-term dialysis or death referred to either the starting date of long-term dialysis or death, whichever came first. The first event of hospitalization associated with a diagnosis of hyperkalemia (ICD-9 code 276.7) during observation period was defined as hyperkalemia-associated hospitalization.

### Statistical analysis

Baseline characteristics were compared by one-way ANOVA for continuous variables and Chi-Square test for categorical variables. In the multivariate Cox proportional hazards regression models, the effects of ACEI, ARB, ACEI+ARB, or ACEIs/ARBs were further adjusted for age, sex, Charlson comorbidity index (CCI), diabetes mellitus, coronary artery disease, stroke, cancer, frequency of visits to nephrologists within 3 years before the index date (0, 1–6,or >6 visits), geographic location (northern, middle, southern, or eastern/other islands, according to NHI registration location), and types of non-ACEI/non-ARB anti-hypertensive agents used. Study entry was defined as the index date. Results were expressed as hazard ratios (HRs) compared with ARB user. The proportional hazards assumption, the constant HR over time, was evaluated by comparing the estimated log-log survival curves for all time-independent covariates. All the log-log survival plots assessed graphically showed 2 parallel lines, indicating no violation of the assumption. Adjusted HRs for long-term dialysis and the composite outcome associated with ACEI/ARB use were further analyzed among subgroups based on participants’ characteristics (see below). The cumulative hazards of long-term dialysis and the composite outcome over time were compared among the ACEI, ARB, ACEI/ARB and ACEI + ARB groups by using the Nelson-Aalen method to adjust covariates adopted in the Cox proportional hazards regression models. All P values were 2-sided, and the level of significance was set at .05. Analyses were performed using commercially available software (SAS, version 9.2 [SAS Institute Inc.], and Stata SE, version 11.0 [Stata Corp]).

## Results

### Characteristics of the study population

**Fig 1:**
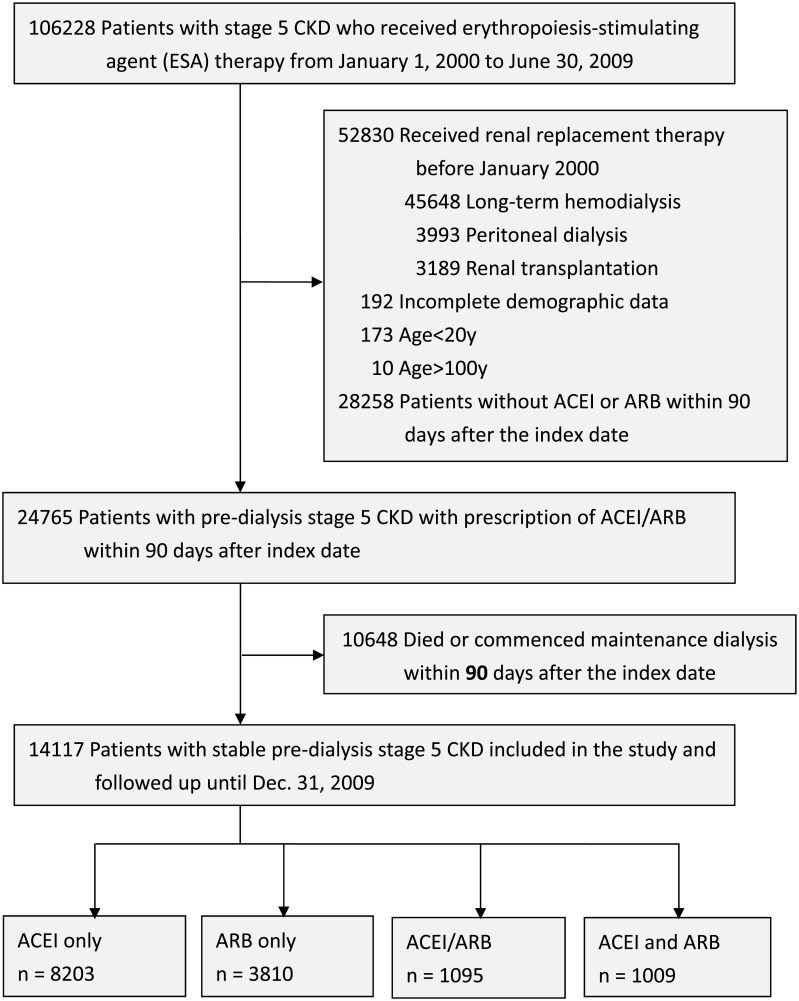
Flowchart of patient enrollment.

We identified 24,765 pre-dialytic advanced CKD patients between January 1, 2000 and January 31, 2009 who met the inclusion criteria. Among them, 10,648 patients died or commenced chronic dialysis within 90 days after the index date. The remaining 14,117 patients were enrolled into the study and classified into four groups as ARB only (N = 8,203, 58.1%), ACEI only (N = 3,810, 26.9%), ACEI/ARB (N = 1,095, 7.7%) and concurrent ACEI+ARB use (N = 1,009, 7.1%) according to their drug prescriptions (Fig 1). The mean age of each group was 64.5, 65.0, 64.2, and 65.0 years, respectively. The distribution of these groups was slightly female (53.4%) and elderly (> = 65 years old; 54.3%) predominant. Compared with ARB user, ACEI user had similar comorbid conditions and medication prescription. However, concurrent ACEI+ARB users, as compared with ARB users, were less comorbid with Diabetes (29.3% vs 58.8%), but more with cardiovascular comorbidities such as myocardial infarction, congestive heart failure and stroke. Concurrent ACEI+ARB users also took more other antihypertensive agents such as alpha-blocker, beta-blocker, calcium channel blocker and diuretics, sodium bicarbonate and potassium lowering agent, compared with users of either ARB or ACEI. (Table 1)

**Table 1:** Baseline characteristics of pre-dialysis stage 5 CKD patients, by treatment options.

Treatment	ARB only	ACEI only	ACEI /ARB	ACEI and ARB	P value
	N = 8,203	N = 3,810	N = 1,095	N = 1,009	
Age, mean (SD), y	64.5 (12.9)	65.0(13.3)	64.2 (13.1)	65.0 (13.5)	
Age, group, y					0.03
20–44	583 (7.1)	288 (7.6)	73 (6.7)	88 (8.7)	
45–64	3,219 (39.2)	1,404 (36.9)	436 (39.8)	361 (35.8)	
65–74	2,423 (29.5)	1,105 (29)	326 (29.8)	297 (29.4)	
75–100	1,978 (24.1)	1,013 (26.6)	260 (23.7)	263 (26.1)	
Gender					0.17
Male	3,786 (46.2)	1,832 (48.1)	495 (45.2)	464 (46)	
Comorbid conditions within 3 y before the index date					
Diabetes	4,826 (58.8)	2,000 (52.5)	315 (28.8)	296 (29.3)	<0.01
MI	2,100 (25.6)	969 (25.4)	673 (61.5)	649 (64.3)	<0.01
CHF	1,117 (13.6)	547 (14.4)	178 (16.3)	179 (17.7)	<0.01
AF	162 (2)	85 (2.2)	24 (2.2)	20 (2)	0.81
Stroke	1,536 (18.7)	704 (18.5)	217 (19.8)	253 (25.1)	<0.01
PAOD	108 (1.3)	54 (1.4)	14 (1.3)	20 (2)	0.39
Cancer	658 (8)	353 (9.3)	91 (8.3)	70 (6.9)	0.05
Charlson Comorbidity Index score					<0.01
<3	2,887 (35.2)	1,451 (38.1)	358 (32.7)	314 (31.1)	
4–5	3,143 (38.3)	1,246 (32.7)	385 (35.2)	363 (36)	
>5	2,173 (26.5)	1,113 (29.2)	352 (32.2)	332 (32.9)	
Mean(SD)	4.4 (2.2)	4.4 (2.4)	4.6 (2.3)	4.7 (2.3)	
Nephrologist visits within 3 y before the index date					<0.01
0	1,400 (17.1)	987 (25.9)	271 (24.8)	212 (21)	
1–6	1,958 (23.9)	1,093 (28.7)	365 (33.3)	217 (21.5)	
>6	4,845 (59.1)	1,730 (45.4)	459 (41.9)	580 (57.5)	
Anti-hypertensive drugs used					
Alpha-blockers	1,824 (22.2)	897 (23.5)	284 (25.9)	322 (31.9)	<0.01
Beta-blockers	3,489 (42.5)	1,478 (38.8)	566 (51.7)	520 (51.5)	<0.01
Calcium channel blockers					
Non-DHP	780 (9.5)	464 (12.2)	166 (15.2)	264 (26.2)	<0.01
DHP	5,768 (70.3)	2,509 (65.9)	829 (75.7)	778 (77.1)	<0.01
Diuretics					
Thiazides	804 (9.8)	356 (9.3)	143 (13.1)	137 (13.6)	<0.01
Loop diuretics	4,967 (60.6)	2,219 (58.2)	757 (69.1)	668 (66.2)	<0.01
Other Anti-hypertensive drugs	862 (10.5)	443 (11.6)	202 (18.5)	233 (23.1)	<0.01
NaHCO3	1,249 (15.2)	675 (17.7)	202 (18.5)	267 (26.5)	<0.01
Calcium polystyrene sulfonate or sodium polystyrene sulfonate	1,622 (19.8)	717 (18.8)	213 (19.5)	345 (34.2)	<0.01
Geographic location in Taiwan					<0.01
Northern	3,627 (44.2)	1,391 (36.5)	300 (27.4)	343 (34)	
Middle	1,820 (22.2)	1,048 (27.5)	332 (30.3)	175 (17.3)	
Southern	2,521 (30.7)	1,301 (34.2)	436 (39.8)	479 (47.5)	
Eastern or other islands	235 (2.9)	70 (1.8)	27 (2.5)	12 (1.2)	

### Renoprotective effects of ACEIs/ARBs in patients with predialysis advanced CKD

**Fig 2:**
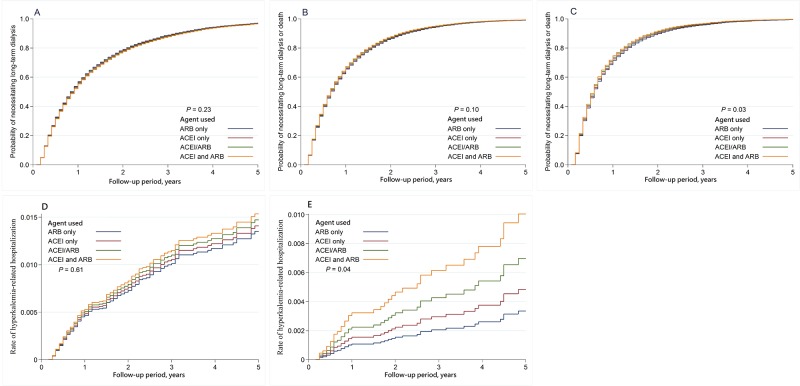
The adjusted cumulative hazards of clinical outcomes. A: The adjusted cumulative hazards of long-term dialysis. B: The adjusted cumulative hazards of long-term dialysis or death. C: The adjusted cumulative hazards of long-term dialysis or death in diabetic sub-group. D: The adjusted cumulative hazards of hyperkalemia associated hospitalization. E: The adjusted cumulative hazards of hyperkalemia associated hospitalization in non-diabetic subgroup.

In a median follow-up of 7 months, 9,867 patients (69.9%) required long-term dialysis and 2,805 (19.9%) died before progression to end-stage renal disease requiring dialysis. The incidence rates of long-term dialysis, death or the composite outcome of dialysis or death are listed in Table 2. The adjusted cumulative hazards of long-term dialysis (Fig 2A) and dialysis or death (Fig 2B) were illustrated as Nelson-Aalen curves for each treatment groups compared with ARB treatment group. The risk of long-term dialysis or the composite outcome of long-term dialysis or death was not significantly different between any two groups. In comparison with ARB only group, however, the risk of death was significantly higher in both ACEI only group (aHR = 1.17, [95%CI, 1.07–1.27]; P = 0.03) and dual blockade group with concurrent use of ACEI and ARB (aHR = 1.49, [95%CI, 1.30–1.71]; P = 0.02)

**Table 2:** Risk of chronic dialysis, dialysis or death, and hyperkalemia associated hospitalization in pre-dialysis stage 5 CKD subjects using ACEI/ARB treatment.

	Subjects	Incidence rate (per 100 person-years)				Adjusted HR (95% CI)			
ACEI/ARB		Dialysis	Death	Dialysis or death	Hyper-kalemia	Dialysis	Death	Dialysis or death	Hyperkalemia
All									
ARB only	8,203	72.5	17.6	90.1	0.42	1.0 (ref.)	1.0 (ref.)	1.0 (ref.)	1.0 (ref.)
ACEI only	3,810	63.8	21.5	85.3	0.30	0.96 (0.92–1.01)	1.17 (1.07–1.27) *	1.01(0.96–1.04)	0.76 (0.52–1.10)
ACEI /ARB	1,095	75.9	21.6	97.5	0.47	1.02 (0.94–1.10)	1.10 (0.95–1.27)	1.03 (0.97–1.10)	0.96 (0.57–1.64)
ACEI and ARB	1,009	71.1	32.4	103.5	0.68	0.95 (0.87–1.04)	1.49 (1.30–1.71) **	1.07 (0.99–1.15)	1.41 (0.84–2.35)
With DM									
ARB only	4,826	84.2	20.4	104.6	0.63	1.0 (ref.)	1.0 (ref.)	1.0 (ref.)	1.0 (ref.)
ACEI only	2,000	73.1	29.5	102.6	0.46	0.96 (0.90–1.02)	1.32 (1.18–1.48) *	1.03 (0.98–1.09)	0.74 (0.47–1.14)
ACEI /ARB	673	86.8	26.8	113.6	0.54	1.02 (0.93–1.13)	1.17 (0.98–1.40)	1.05 (0.96–1.14)	0.76 (0.39–1.48)
ACEI and ARB	649	81.4	40.2	121.6	0.81	0.96 (0.86–1.07)	1.58 (1.34–1.86) **	1.10 (1.01–1.2) *	1.13 (0.61–2.09)
Without DM									
ARB only	3,377	60.9	14.8	75.7	0.19	1.0 (ref.)	1.0 (ref.)	1.0 (ref.)	1.0 (ref.)
ACEI only	1,810	56.7	15.6	72.3	0.18	0.96 (0.90–1.03)	0.99 (0.86–1.13)	0.97 (0.91–1.03)	0.79 (0.39–1.58)
ACEI /ARB	422	63.9	15.9	79.8	0.38	1.01 (0.90–1.14)	1.02 (0.80–1.30)	1.01 (0.91–1.13)	1.77 (0.70–4.44)
ACEI and ARB	360	47.9	18.7	66.6	0.51	0.95 (0.82–1.09)	1.46 (1.16–1.85) *	1.06 (0.94–1.19)	2.74 (1.05–7.15) +

^+^p value = 0.04,

*p value = 0.03,

** *p* value = 0.02,

IR: incidence rate, per 100 person-years.

Multivariate analysis was adjusted for variables as listed in Table 1, ref.: reference

When patients were stratified into diabetic and non-diabetic subgroups by taking the status of the patients treated with ARB only as reference index, dual blockade with concurrent use of ACEI and ARB was associated with a significantly higher risk of the composite outcome of long-term dialysis or death only in diabetic subgroup (Fig 2C, aHR = 1.10, [95%CI, 1.01–1.20]; P = 0.03), but not in non-diabetic subgroup (aHR = 1.06, [95%CI, 0.94–1.19]). In the diabetic subgroup, the risk of death was significantly higher in both ACEI only users (aHR = 1.32, [95%CI, 1.18–1.48]; P = 0.03) and users of dual blockade with ACEI and ARB (aHR = 1.58, [95%CI, 1.34–1.86]; P = 0.02). However, in the non-diabetic subgroup, the risk of death was significantly higher in users of dual blockade with ACEI and ARB (aHR = 1.46, [95%CI, 1.16–1.85]; P = 0.03) but not in ACEI only users (aHR = 0.99, [95%CI, 0.86–1.13]). In terms of the risk of long-term dialysis, there was no significantly difference between any groups of different RAS blockade users in either diabetic or non-diabetic patients.

When patients were stratified by time period as pre-dialysis and under dialysis subgroups as shown in S1 and S2 Tables. In comparison with ARB only users, ACEI only users and users of dual blockade with ACEI and ARB remained associated with a significantly higher risk of death after dialysis in all patients (aHR = 1.61; 1.83 vs. 1), in DM subgroup (aHR = 1.75; 1.76 vs.1) as well as in non-DM subgroup (aHR = 1.41; 2.18 vs. 1) (S2 Table). In addition, as compared with ARB-only users, the risk of death (aHR) after dialysis was higher than that before dialysis for ACEI-only users, not only in all patients (1.61 vs. 1.17) but also in non-DM subgroup (1.41 vs. 0.99) as well as DM subgroup (1.75 vs. 1.31).

### Hyperkalemia-related hospitalization

Compared with ARB users, concurrent ACEI+ARB users showed to run a higher, but not to a significant level, risk of hyperkalemia-associated hospitalization (aHR of 1.41; 95% CI, 0.84–2.35). (Table 2, Fig 2D) However, when we stratified patients according to their status of diabetes, dual blockade was associated with a significantly higher risk of hyperkalemia- associated hospitalization in non-diabetic CKD patients (HR, 2.74, [95%CI, 1.05–7.15]; P = 0.04, Fig 2E) but not in diabetic patients (HR = 1.13, [95%CI, 0.61–2.09]).

## Discussion

Our study demonstrated that dual blockade therapy with ACEIs and ARBs in predialysis CKD patients was not significantly associated with long-term dialysis or death. Dual RAS blockade has been thought to be more renoprotective than monotherapy because of its effect on proteinuria reduction in the short-term treatment.[8] However, some large clinical trials such as ONTARGET[6] have otherwise disclosed that dual RAS blockade, instead of being effective, might be harmful because of its potential to increase the risk of acute kidney injury, cardiovascular events and hyperkalemia. They thus excluded CKD patients with serum creatinine above 3 mg/dl from the study. Another randomized control trial, the Veterans Affairs Nephropathy in Diabetes (VA NEPHRON-D) study, also showed no significant survival benefit but increased risk of hyperkalemia and acute kidney injury with combination therapy than with monotherapy by enrolling 1,448 diabetic nephropathy patient with CKD stage 1–3, an estimated glomerular filtration rate (GFR) of 30.0 to 89.9 ml per minute per 1.73 m^2^.[9] To our knowledge, there has been no investigation published to evidence the superior safety and effectiveness of dual RAS blockade to monotherapy in advanced CKD patients. The current study might fill this gap.

In this study, the subgroup analysis disclosed a 10% higher risk of long-term dialysis or death among diabetic patients. The different effect of dual RAS blockade and monotherapy between diabetic and non-diabetic patient has also been reported by Kim et al. in their small population crossover trial.[10] In their trial, 24 diabetic nephropathy patients and 19 non-diabetic renal diseases (IgA nephropathy) patients received a crossover treatment with ramipril and dual RAS blockade (Ramipril and Candesartan) in 12-week period. Compared with monotherapy, this intervention seemed to significantly reduce the 24-hour urinary protein excretion rate in non-diabetic renal disease patients, but made little difference in diabetic patients. Another prospective crossover trial enrolling 14 patients with IgA nephropathy and 18 with type 2 diabetic CKD conducted by Song et al. revealed that the urinary TGF-beta1 excretion reduced with dual RAS blockade, compared with monotherapy, only in IgA nephropathy patients,[11] not in the diabetic ones. The possible explanations for this difference include the pathophysiological mechanism of diabetic nephropathy, which is more heterogeneous and complex than non-diabetic renal disease, and several other factors such as oxidative stress, glycation end-products, and micro-inflammatory response other than intraglomerular hypertension and RAS activation.[12]

Few studies were focused on the effect of dual RAS blockade to non-diabetic CKD patient group. A former Clinical trial on Aliskiren in Type 2 Diabetes and Cardiorenal End Points (ALTITUDE) choosing the direct renin inhibitor Aliskiren, instead of ACEI, as the second RAS blockade added on ARB in diabetic patients was terminated early because of increasing adverse events including stroke, hyperkalemia and renal complications.[13] On the other hand, another investigation in similar study design using dual blockade combining direct renin inhibitor plus ARB, compared with ARB monotherapy, benefited the non-diabetic CKD patient significantly by reducing their proteinuria and slowing renal function decline.[14] In our study, although more comorbidities, including myocardial infarction, congestive heart failure, and stroke, were observed in ACEI plus ARB group than in monotherapy group (Table 1), the outcome of death or long-term dialysis did not make significant difference to non-diabetic CKD patients. It is worthy to perform further investigation to identify if non-diabetic CKD patients could tolerate and benefit from dual RAS blockade more than from monotherapy.

Our finding of increased risk of death not only in dual blockade users but also in ACEI users as compared with ARB users was supported by the report of Chan et al. who compared the relative effectiveness of newly add-on ACEIs, ARBs and dual blockades in reducing cardiovascular mortality in about 9,300 chronic hemodialysis (HD) patients over 6 years.[15] After adjustment for risk factors, increased risk of cardiovascular death was observed in 6,866 patients on ACEI with non-ARB antihypertensive agent (HR of 1.27) as well as in 701 patients initiated on combined ACEI and ARB therapy (HR of 1.45) as compared with 1,758 patients initiated on an ARB with non-ACEI antihypertensive therapy.

The reason why ACEIs may be associated with higher risk of death could be explained by the following mechanisms. First, the anti-inflammatory effect of ACEIs may be less potent than ARBs according to the report by Gamboa et al.[16], who conducted a randomized, double-blind 3×3 crossover study of 15 HD patients assigned to placebo, ACEI with ramipril (5 mg/d), and ARB with valsartan (160 mg/d) for 7 days. Although valsartan and ramipril both lowered interleukin-6 (IL-6) levels during dialysis, ramipril increased IL-1β concentrations and decreased IL-10 concentrations compared with placebo, leading to the conclusion that ARB may induce a greater anti-inflammatory effect than ACEI. Second, ACEIs may aggravate endothelial dysfunction by increasing the plasma levels of asymmetric dimethylarginine (ADMA) in HD patients, as it was demonstrated in a randomized cross-over study by Gamboa et al.[17] They found that ADMA levels were significantly increased throughout the dialysis session during the treatment by ramipril as compared with valsartan or placebo. Furthermore, they also showed that ACE inhibition increased bradykinin (BK) levels in ESRD patients during HD. The mechanism could be explained by the in vitro study that showed the incubation with BK increased intracellular ADMA concentration through the stimulation of BK B2-receptor in A549 cells.[17]

The inflammatory status in the period after dialysis should be more severe than that before dialysis because of less excretion of pro-inflammatory cytokines due to lower residual renal function. Furthermore, DM patients are also associated with higher inflammatory status than non-DM patients. Thus, the weaker anti-inflammatory effect by ACEI (vs. ARB) may be associated with a higher risk of death in either study period (after vs. before dialysis) or patient subgroup (DM vs. non-DM) with more severe inflammation.

Hyperkalemia was one of the most concerned adverse effects of RAS blockade, either ACEIs or ARBs. The combination therapy with ACEI plus ARB exhibits even more complete blockade of RAS capable to elevate serum potassium level to a much higher level than ACEI or ARB monotherapy can in CKD patients.[18] A previous retrospective analysis of 245,808 patients have reported that RAS blockade increased the risk of hyperkalemia more in patients with CKD than in those without CKD (7.67 vs. 2.30 per 100 patient months, p<0.0001). However, the odds ratio of death from a moderate (K+≥ 5.5 and < 6.0mg/dl) to severe (K+≥ 6.0mg/dl) hyperkalemia event was significantly higher in non-CKD patient than in CKD patients.[19] CKD patients, compared with normal population, could tolerate hyperkalemia more and might not exhibit apparent electrocardiographic or cardiovascular manifestations. Jung Nam An et al. has reported an observational cohort study for 923 patients, including 70.2% CKD and 40.6% DM patients who were hospitalized due to severe hyperkalemia, to observe a lower mortality rate in patients with diabetes and CKD. Furthermore, as CKD progressed to higher stages, the OR of in-hospital mortality decreased.[20] This may help explain why the risk of hyperkalemia-associated hospitalization increased so significantly with combination therapy than with monotherapy in our non-diabetic CKD subgroup, but not in diabetic CKD patients. Distinct from non-diabetic CKD, diabetic nephropathy usually exhibits chronic hyperkalemia as a result of hyporeninemic hypoaldosteronism, which suppresses potassium secretion from renal tubule.[21] Dual RAS blockade may not precipitate hyperkalemia in these patients so strong as in those CKD patients without hyporeninemic hypoaldosteronism syndrome.

The major strengths of our study are the large sample size and its nationally representative nature, which rely on a comprehensive medical utilization claim data base to include all the CKD patients as possible. Bias on the date of immortality was eliminated as best as can by selecting patients who survived beyond 90 days after ESA prescription and could be observed thereafter.[22] However, several limitations of this study should be addressed. First, the diagnosis of advanced CKD came from the administrative claims data and might not be made as accurate as by clinically standard practice. For example, patients reported with acute kidney injury on top of chronic kidney injury may exhibit transient elevation of creatinine level to >6 mg/dl. Thus, we restricted the analyses to the patients receiving ESA therapy for at least 2 ambulatory care visits to minimize the bias. Second, the onset of pre-dialytic stage 5 CKD in this study was defined as the first day of ESA prescription. For those patients who sought medical assistance late or sought alternative treatment, the timing of enrollment may vary and introduce bias. Third, blood pressure level was not available from database and we analyzed different kinds of antihypertensive agents use in each group. Although more antihypertensive agents prescribed in dual RAS blockade users may introduce bias due to poor blood pressure control, ARB only users, who showed no difference in other antihypertensive agents use from ACEI only users, still associated with lower mortality. Fourth, the information on several potential confounders of mortality and hyperkalemia episodes, such as base-line creatinine and potassium level, severity of proteinuria, body-mass index and so on, were not available from the database. Finally, the present study enrolled pre-dialytic stage 5 CKD patients who were receiving ESA treatment. These results may not be extrapolated to all patients with pre-dialytic CKD.

## Conclusion

Our study demonstrates the difference in the effectiveness and safety between ACEIs, ARBs and dual RAS blockade therapy (ACEI plus ARB) in pre-dialytic stage 5 CKD patients. As compared with ARBs, dual blockade with ACEIs and ARBs shows a higher risk of death and composite endpoint of long-term dialysis or death in diabetic subgroup as well as a higher rate of hyperkalemia-associated hospitalization in non-diabetic subgroup, and ACEIs are associated with a higher risk of death, especially in diabetic subgroup. Thus, monotherapy of RAS blockade, especially ARB, is more effective and safer than dual RAS blockade in pre-dialytic stage 5 CKD patients.

## Supporting information

S1 Table Risk of death before dialysis initiation in pre-dialysis stage 5 CKD subjects using ACEI/ARB treatment. (DOCX)

S2 Table Risk of death after dialysis initiation in pre-dialysis stage 5 CKD subjects using ACEI/ARB treatment. (DOCX)

S3 Table Diagnostic codes for chronic kidney disease and comorbid conditions. (DOCX)
