# Supplementary material for: Angiotensin receptor blockers are associated with lower mortality than ACE inhibitors in predialytic stage 5 chronic kidney disease: A nationwide study of therapy with renin-angiotensin system blockade
Source: PLoS One. 2017 Dec 7;12(12):e0189126. doi: 10.1371/journal.pone.0189126 (PMC5720519; doi:10.1371/journal.pone.0189126)
Supplement: S1 Table — (DOCX) [file pone.0189126.s001.docx]

**S1 Table. Risk of death before dialysis initiation in pre-dialysis stage 5 CKD subjects using ACEI/ARB treatment**

| ACEI/ARB | Subjects (N) | Pre-dialysis Death (n) | Pre-dialysis mortality rate (per 100 person-years) | Crude HR (95%CI) | Adjusted HR (95%CI) |
| --- | --- | --- | --- | --- | --- |
| All |  |  |  |  |  |
| ARB only | 8,203 | 1,425 | 18.79 | 1.0 (ref.) | 1.0 (ref.) |
| ACEI only | 3,810 | 888 | 22.80 | 1.23(1.13-1.33)^**^ | 1.17(1.07-1.27)^**^ |
| ACEI /ARB | 1,095 | 218 | 23.72 | 1.25(1.09-1.44)^**^ | 1.11(0.96-1.29) |
| ACEI and ARB | 1,009 | 274 | 37.48 | 1.98(1.74-2.26)^**^ | 1.56(1.37-1.79)^**^ |
| With DM |  |  |  |  |  |
| ARB only | 4,826 | 828 | 22.46 | 1.0 (ref.) | 1.0 (ref.) |
| ACEI only | 2,000 | 523 | 32.32 | 1.42(1.28-1.59)^**^ | 1.31(1.17-1.47)^**^ |
| ACEI /ARB | 673 | 142 | 30.47 | 1.36(1.13-1.62)^**^ | 1.19(1.00-1.43) |
| ACEI and ARB | 649 | 187 | 48.57 | 2.18(1.86-2.56)^**^ | 1.65(1.40-1.94)^**^ |
| Without DM |  |  |  |  |  |
| ARB only | 3,377 | 597 | 15.32 | 1.0 (ref.) | 1.0 (ref.) |
| ACEI only | 1,810 | 365 | 16.03 | 1.06(0.93-1.21) | 0.99(0.87-1.13) |
| ACEI /ARB | 422 | 76 | 16.78 | 1.08(0.85-1.37) | 1.00(0.79-1.28) |
| ACEI and ARB | 360 | 87 | 25.14 | 1.62(1.29-2.03)^**^ | 1.53(1.21-1.94)^**^ |

**p* value < 0.05, ** *p* value < 0.01, IR: incidence rate, per 100 person-years.

Multivariate analysis was adjusted for variables as listed in Table 1, ref.: reference
