# Supplementary material for: Angiotensin receptor blockers are associated with lower mortality than ACE inhibitors in predialytic stage 5 chronic kidney disease: A nationwide study of therapy with renin-angiotensin system blockade
Source: PLoS One. 2017 Dec 7;12(12):e0189126. doi: 10.1371/journal.pone.0189126 (PMC5720519; doi:10.1371/journal.pone.0189126)
Supplement: S2 Table — (DOCX) [file pone.0189126.s002.docx]

**S2 Table. Risk of death after dialysis initiation in pre-dialysis stage 5 CKD subjects using ACEI/ARB treatment**

| ACEI/ARB | Subjects (N)  Under dialysis | Death after dialysis (n) | Post-dialysis mortality rate (per 100 person-years) | Crude HR (95%CI) | Adjusted HR (95%CI) |
| --- | --- | --- | --- | --- | --- |
| All |  |  |  |  |  |
| ARB only | 6,433 | 1,015 | 17.12 | 1.0 (ref.) | 1.0 (ref.) |
| ACEI only | 2,785 | 701 | 24.21 | 1.50(1.36-1.65)^**^ | 1.61(1.46-1.78)^**^ |
| ACEI /ARB | 839 | 170 | 24.29 | 1.40(1.19-1.65)^**^ | 1.26(1.07-1.48)^**^ |
| ACEI and ARB | 670 | 165 | 33.40 | 2.02(1.71-2.38)^**^ | 1.83(1.55-2.17)^**^ |
| With DM |  |  |  |  |  |
| ARB only | 3,775 | 687 | 24.57 | 1.0 (ref.) | 1.0 (ref.) |
| ACEI only | 1,397 | 439 | 40.80 | 1.73(1.53-1.95)^**^ | 1.75(1.55-1.98)^**^ |
| ACEI /ARB | 505 | 121 | 36.23 | 1.45(1.20-1.76)^**^ | 1.32(1.08-1.60)^**^ |
| ACEI and ARB | 427 | 115 | 48.52 | 2.06(1.69-2.51)^**^ | 1.76(1.44-2.16)^**^ |
| Without DM |  |  |  |  |  |
| ARB only | 2,658 | 328 | 10.47 | 1.0 (ref.) | 1.0 (ref.) |
| ACEI only | 1,388 | 262 | 14.4 | 1.43(1.21-1.68)^**^ | 1.41(1.20-1.67)^**^ |
| ACEI /ARB | 334 | 49 | 13.39 | 1.28(0.95-1.73) | 1.10(0.81-1.50) |
| ACEI and ARB | 243 | 50 | 19.46 | 1.90(1.41-2.55)^**^ | 2.18(1.61-2.96)^**^ |

**p* value < 0.05, ** *p* value < 0.01, IR: incidence rate, per 100 person-years.

Multivariate analysis was adjusted for variables as listed in Table 1, ref.: reference
