# Supplementary material for: Angiotensin receptor blockers are associated with lower mortality than ACE inhibitors in predialytic stage 5 chronic kidney disease: A nationwide study of therapy with renin-angiotensin system blockade
Source: PLoS One. 2017 Dec 7;12(12):e0189126. doi: 10.1371/journal.pone.0189126 (PMC5720519; doi:10.1371/journal.pone.0189126)
Supplement: S3 Table — (DOCX) [file pone.0189126.s003.docx]

**S3 Table. Diagnostic codes for chronic kidney disease and comorbid conditions**

| **Disease** | **ICD-9-CM codes** |
| --- | --- |
| **Chronic kidney disease** | 016.0x, 042, 095.4, 189.x, 223.x, 236.9, 250.4x, 271.4, 274.1x, 403.x-404.x, 440.1, 442.1, 446.21, 447.3, 572.4, 580.x-589.x, 590.x-591, 593.x, 642.1x, 646.2x, 753.x, 984.x |
| **Comorbidities** |  |
| Diabetes mellitus | 250.x |
| Myocardial infarction | 410.x |
| History of heart failure | 428.x |
| Atrial fibrillation | 427.31 |
| History of stroke | 430.x – 438.x |
| Peripheral artery occlusive disease | 440.x, 443.x, 444.2x, 444.8x, 444.9, 447.8, 447.9 |
| Cancer | 140.x – 208.x |
